# Lateral Flow Assays for the Diagnosis of Invasive Aspergillosis: Current Status

**DOI:** 10.1007/s12281-017-0275-8

**Published:** 2017-04-29

**Authors:** Sven Heldt, Martin Hoenigl

**Affiliations:** 1 Division of Pulmonology, Medical University of Graz, Graz, Austria; 2 Section of Infectious Diseases and Tropical Medicine, Medical University of Graz, Auenbruggerplatz 15, 8036 Graz, Austria; 3 CBmed—Center for Biomarker Research in Medicine, Graz, Austria; 4 Division of Infectious Diseases, Department of Medicine, University of California–San Diego, San Diego, USA

**Keywords:** Aspergillus lateral flow device test, Point of care, Galactomannan-like antigens, MAb476, JF5, Bronchoalveolar lavage, Urine, Serum, Monoclonal antibody, Invasive aspergillosis

## Abstract

**Purpose of Review:**

Diagnosis during early stages of invasive aspergillosis (IA) and targeted antifungal treatment has the potential to improve survival significantly. Despite advances in the diagnostic arsenal, invasive mold infections remain difficult to diagnose—especially at early stages before typical radiological signs develop. Varying availability and time-to-results are important limitations of current approved biomarkers and molecular assays for diagnosis of IA. Here, we will give an update on the *Aspergillus*-specific lateral-flow device (LFD) test. We further review promising findings on feasibility of point-of-care (POC) detection of urinary excreted fungal galactomannan-like antigens.

**Recent Findings:**

POC LFD assays for detection of *Aspergillus* antigens are currently in development. The *Aspergillus*-specific LFD test, which is based on the JF5 antibody (Ab), detects an extracellular glycoprotein antigen secreted during active growth of *Aspergillus* spp. The test has shown promising results in various studies. In addition, a monoclonal Ab476-based LFD for POC detection of urinary excreted fungal galactomannan-like antigens has been developed but needs further validation.

**Summary:**

Important advances have been made in the development of LFD assays for IA. Most promising is the *Aspergillus*-specific LFD test; commercial availability is still pending, however. The search for reliable POC tests for other molds, including mucorales, continues.

## Introduction

Invasive aspergillosis (IA) is associated with high mortality rates [1–5]. Early and reliable diagnosis and rapid initiation of appropriate antifungal therapy has been shown to improve survival significantly [6, 7]. Culture-based approaches are important for detection of fungal species and resistance testing; however, they are limited by low sensitivities—in particular during early phases of infection—and long turnaround time [8•]. Significant advances to the field were brought by the introduction of non-cultural diagnostic tests for IA in blood and bronchoalveolar lavage fluid (BALF), including galactomannan antigen (GM) testing [9•, 10 –13], PCR [14••, 15, 16••, 17], and beta-d-glucan (BDG) testing [18–24] in patients at risk [25•, 26, 27, 28•, 29]. In line with the introduction of non-cultural diagnostic tests, the rate of fungal infections diagnosed pre-mortem (versus post-mortem) was shown to increase from 16 to 51% in a large autopsy study [30].

Despite these significant advancements, the availability of these non-cultural diagnostic tests and time-to-results often varies with the size, specialization, and resources of the medical institution. “Pregnancy test-like” point-of-care lateral flow assays for detection of *Aspergillus* antigens are currently in development and may overcome these limitations.

### *Aspergillus*-Specific Lateral Flow Device Test

The point-of-care *Aspergillus*-specific lateral-flow device test (LFD) uses a mouse monoclonal antibody, JF5, which binds to an extracellular glycoprotein antigen secreted by *Aspergillus* spp. only during active growth [31]. The LFD can be used for testing of serum and BALF samples and shows cross-reaction with *Penicillium* spp. only [31]. While serum samples require heating, centrifugation, and addition of a buffer solution before testing, BALF samples can be tested without any pre-treatment [32]. After 15 min of incubation time, results are read by the naked eye and are interpreted depending on the intensity of the test line as negative (−) or weak (+) to strong (+++) positive and have been shown to be reproducible between laboratories and studies [33, 34].

To date, two studies evaluated the diagnostic performance of the LFD in serum samples from adult patients with hematological malignancies [14, 35•], reporting sensitivities of 40 and 82% and specificities 87 and 80% for probable/proven invasive aspergillosis (IA) according to modified EORTC/MSG criteria [36], respectively. A meta-analysis, which included also data from the LFD development study [31] (i.e., in addition to the two studies mentioned above), reported a pooled sensitivity of 68% (95% confidence interval (CI), 52–81%), specificity of 87% (95% CI, 80–92%), and diagnostic odds ratio (DOR) of 11.90 (95% CI, 3.54–39.96) for differentiating proven/probable versus no IA cases in serum samples [37•]. Another very recently published study ignored the recommendations of the manufacturer by using the LFD in serum samples without pretreatment and found poor performance confirming that pretreatment of serum samples is a necessary step and that recommendations of the manufacturer should be followed [38]. Overall, the requirement for pretreatment has been a major limitation of serum LFD testing, as has been the inconsistency of reported results. Further studies, including multicenter studies, are needed to determine whether LFD serum testing can be recommended for clinical routine.

In contrast to serum testing, BALF LFD testing has been evaluated in a number of studies including multicenter studies and in different patient populations. Results from the first four-part, retrospective-part, prospective studies (including two multicenter studies) which evaluated the LFD in mostly patients with underlying respiratory diseases [39••] and solid organ transplant recipients [28, 40••, 41] but also a smaller proportion of patients with underlying hematological malignancies [28, 41], were summarized in the meta-analysis reporting a pooled sensitivity of 86% (95% CI, 76–93%), specificity of 93% (95% CI, 89–96%), and DOR of 65.94 (95% CI, 27.21–159.81) for IA when using BALF samples [37].

**Fig. 1:**
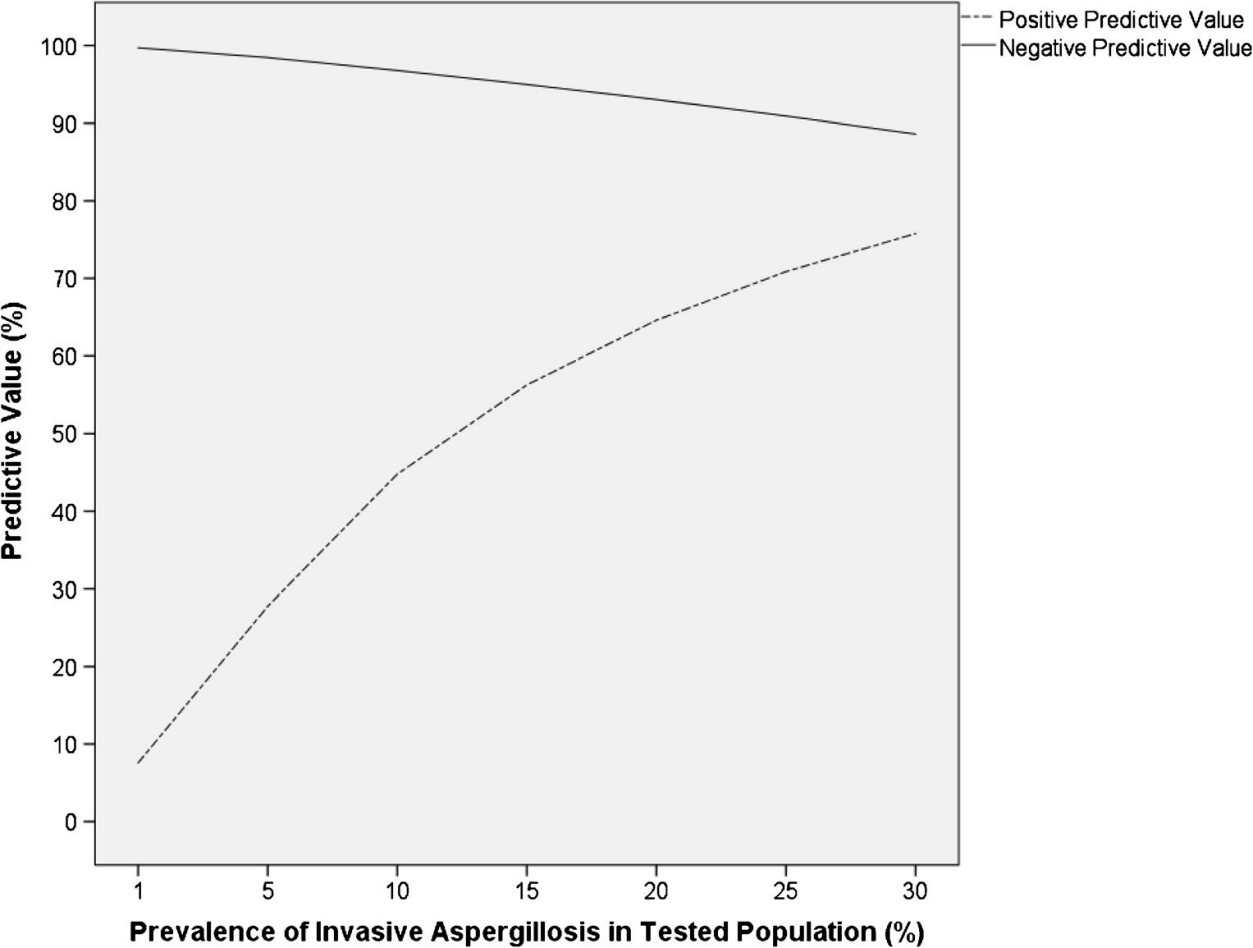
Overall positive and negative predictive values of the bronchoalveolar lavage fluid *Aspergillus*-specific lateral flow device test in cohorts with prevalence rates of invasive aspergillosis between 1 and 30%. The overall sensitivity of 73% and specificity of 90% were used for the calculation

Since then, BALF LFD testing has been evaluated in multicenter studies in intensive care unit patients [42••] and patients with underlying hematological malignancies [43], as well as a number of single-center studies [16••, 44–47]. The up-to-date performance of the LFD in BALF samples for different patient groups as well as the overall performance per sample are depicted in Table 1. Published data indicates that to date, 792 BALF samples have been tested at 6 different medical universities, including 113 samples from patients with probable/proven IA and 552 samples from patients with no evidence of IA, resulting in an overall sensitivity of 73% and specificity of 90% for probable/proven IA versus no evidence for IA. While the overall positive predictive value (PPV) was 61% and the negative predictive value (NPV) 94% in samples tested to date, both PPV and NPV will depend on the prevalence of IA in tested populations as displayed in Fig. 1. For example, in a patient cohort with an IA prevalence of 1%, the PPV will be 7.6%, while the NPV will be 99.7%; the PPV will go up and the NPV down with increase of IA prevalence (e.g., 5% IA prevalence: PPV 28%, NPV 98.4%; 10% IA prevalence: PPV 45%, NPV 96.8%; 20% IA prevalence: PPV 65%, NPV 93%; always assuming 73% sensitivity and 90% specificity).

**Table 1:** Per BALF sample performance of the BALF *Aspergillus* LFD for probable/proven invasive pulmonary aspergillosis versus no evidence for invasive pulmonary aspergillosis in different patient cohorts (percentage and absolute numbers)^a^

Patient group	Sensitivity	Specificity	PPV	NPV
Overall^b^	73% (83/113)	90% (498/552)	61% (83/137)	94% (498/528)
Solid organ transplantation	94% (15/16)	92% (89/97)	65% (15/23)	99% (89/90)
Intensive care unit	79% (26/33)	85% (176/206)	46% (26/56)	96% (176/183)
Respiratory diseases	78% (25/32)	91% (196/215)	57% (25/44)	97% (196/203)
Hematological malignancies	67% (36/54)	91% (126/139)	73% (36/49)	88% (126/144)

*PPV* positive predictive value, *NPV* negative predictive value

^a^Data derived from published studies [8•, 16••, 28•, 32, 33, 39–47, 48••]:

^b^Overall summarizes unique samples and is lower than the sum of subgroup samples, as some samples were classified into more than one subgroup

Table 2 summarizes LFD results in 127 samples from patients with possible IPA according to modified EORTC/MSG criteria [36, 49], as well as patient subgroups. The LFD resulted positive in 39% of possible IA samples versus 10% of samples from patients with no evidence of IA. When considering that diagnostic test performance calculations in the field of IA are limited by the insensitivity of all current diagnostics (including GM), these results may indicate that the LFD has additional discriminatory power in those with possible IPA, i.e., positive LFD results may provide evidence that some of those with possible IPA do in fact have (probable) IPA and a false negative GM test result. If true, positive BALF LFD test results should be considered as a future mycological factor (in addition to GM, culture, and PCR) for updated EORTC/MSG criteria.

**Table 2:** Performance of the BALF *Aspergillus* LFD in cases of possible invasive pulmonary aspergillosis (per BALF sample) in different patient cohorts.^a^

Patient group	Positive LFD Result Percentage (absolute numbers)	Negative LFD Result Percentage (absolute numbers)
Overall^b^	39% (50/127)	61% (77/127)
*Solid organ transplantation*	*33%* (*4*/*12*)	*66%* (*8*/*12*)
*Intensive care unit*	*37%* (*14*/*38*)	*63%* (*24*/*38*)
*Respiratory diseases*	*65%* (*20*/*31)*	*35%* (*11*/*31*)
*Hematological malignancies*	*32%* (*26*/*81)*	*68%* (*55*/*81*)

^a^Data derived from published studies [16••, 28•, 39•, 40, 41, 42••, 43••, 46, 47]

^b^Overall summarizes unique samples and is lower than the sum of subgroup samples, as some samples were classified into more than one subgroup

When comparing different patient populations (Table 1), LFD sensitivity was lower in patients with underlying hematologic malignancies (67% sensitivity) compared to other patient groups. The most likely reason for lower sensitivity in patients with hematological malignancies is the frequent use of antifungal prophylaxis/empirical antifungal therapy that is beneficiary for survival in this patient population [50–56]. Similar to other fungal diagnostics, sensitivity of the LFD was reduced in the presence of antifungal prophylaxis/treatment (sensitivity 56% in those with mold active antifungals versus 86% in those without; *p* = 0.0097 Fisher’s exact test; Table 3) [8•]. The solution is combining the LFD with other biomarkers such as GM in BALF and/or serum, PCR, or novel biomarkers such as triacetylfusarinine C (TAFC) [14••, 16••, 28, 43••, 45, 48••], which has been shown to increase sensitivity substantially and helps to overcome this limitation.

**Table 3:** Sensitivity of BALF LFD for probable/proven IPA in patients with and without antifungal prophylaxis/therapy (information only available for a proportion of cases published).^a^

	BALF LFD sensitivity for IPA overall percentage (absolute numbers)
Overall	75% (50/67)
Under mold active systemic antifungals	56% (14/25)
Without mold active antifungals	86% (36/42)

*BALF* bronchoalveolar lavage fluid, *IPA* invasive pulmonary aspergillosis, *LFD* lateral flow device

^a^Data derived from [8•], updated with [16••, 45, 46]

After issues emerged with the previous manufacturing partner, redevelopment work was undertaken by OLM diagnostics after it was given full control to develop and manufacture the assay on top of its original role as sales and marketing partner. Development work is well underway and OLM are expecting to start production over the coming months and launch the LFD by the end of 2017.

### Lateral Flow Device for Galactomannan-Like Antigens in Urine

Antigen testing of urine samples may provide important advantages, including non-invasive and easy sample collection that allows for more frequent examination of large volumes, which may increase test sensitivity and also has great potential for home testing [57, 58••]. Recent studies have indicated that GM testing may be promising in urine samples [57, 59, 60], while results for urine BDG testing were less convincing [61]. Fisher and colleagues reported lower specificity of GM testing of urine specimens compared to serum (80% versus 95%) in pediatric hematologic malignancy patients; however, urine GM testing successfully identified the only case of probable IA [60]. These preliminary results were confirmed in a study conducted in adult hematologic malignancy patients by Duettmann and colleagues [62]. In that study, 242 same-day serum and urine samples were included from 75 adult patients prospectively and consecutively. Out of these 75, 10 patients met criteria for probable IA; 3 additional patients were tested positive for serum GM levels. Urine samples were not pretreated before GM testing, and urine GM levels showed a significant positive correlation with serum GM levels. Sensitivity of urine GM testing was limited and only improved when using an extremely low GM cut-off of 0.1 optical density index (ODI). With that cut-off, urine GM testing exhibited 71% sensitivity and 88% specificity for probable IA. Recently, Reischies and colleagues showed in another prospective study in adult patients with hematological malignancies that test performance in urine samples can be improved by calculation of the urine GM/creatinine ratio, which takes urine dilution into account and may be a promising diagnostic tool for patients with hematological malignancies [57]. With a threshold of 0.26 ((urine GM [ODI] × 100)/(urine creatinine [mg/dL])), the positive predictive value of 13% was low, but the negative predictive value of >98% would qualify this diagnostic method for ruling out IA in high-risk patients [57].

Given this promising results, development of a point of care (POC) test for diagnosis of IA in urine samples has the potential of impacting patient care and associated costs significantly, as such a test may allow for home testing for IA. Dufresne and others recently reported that their new monoclonal antibody MAb476 was capable to detect GM-like antigen (Ag) in urine samples [58••]. Using in vitro and animal experiments, Dufresne and others [58••] investigated renal clearance of serum GM-like Ag in mouse models infected with *A. fumigatus* and generated MAb476. MAb476-based sandwich-ELISAs (sELISA) reliably detected GM-like Ag in bronchoalveolar lavage fluid, serum, and lung tissue of neutropenic mice and urine samples from guinea pigs after infection with air-borne IA [58••]. Experiments showed a specific affinity of MAb476 to *Aspergillus* spp. (excluding *A. terreus*) as well as *Fusarium* spp., *Paecilomyces* spp., and *Trichophyton rubrum* [58••]. MAb476 therefore differed from the EBA2 antibody used in Platelia® GM EIA, which showed also affinity to *A. terreus* and *Histoplasma capsulatum* C Ag [58••]. While these two antibodies may show affinity to different epitopes of the GM-like Ag, which would explain the differences, the detailed structure of the GM-like Ag has not yet been revealed [58••].

As a next step, Dufresne and colleagues successfully constructed a MAb476-based LFD prototype for urine POC testing. The functionality of MAb476 testing was confirmed with human urine samples, which were obtained from healthy volunteers and spiked with in vitro-produced *Aspergillus* antigen [58••]. However, when retrospectively testing stored samples which were collected from 11 patients who were categorized as probable/proven IA and showed positive serum GM results, sensitivity was imperfect, as only samples from 4 out of these 11 patients also had positive test results with the MAb476-sELISA and MAb476-LFD after pretreatment [58••]. Importantly, MAb476 testing appeared to be inhibited by an unknown substance or mix of substances in human urine, and this effect positively correlated to the specific mass of the urine samples [58••]. Boiling and centrifugation were not able to abandon the inhibition, and the relevant substances appeared to weigh less than 2 kDa [58••]. Urine sample concentration (5–10 fold), followed by desalting/dialysis (7 kDa), resulted in a nearly complete diminution of the inhibition [58••].

While these results on development of the MAb476-LFD for urine samples were promising, it has to be kept in mind that the sample size was limited and the study settings were non-clinical and animal models in large parts. Additionally, the quality of the clinical samples and other information about the patients (like the presence of antifungal treatment) could not be satisfactorily evaluated [58••]. Referring to the planned use in clinical settings, the MAb476-LFD needs further optimization to fit requirements for a reliable POC device, which is currently ongoing.

## Conclusion

Important advances have been made in the development of lateral flow assays for invasive aspergillosis. Most promising is the *Aspergillus*-specific lateral-flow device test, which is based on the JF5 antibody and has shown convincing performance in multiple clinical studies, in particular in BALF samples. Commercial availability is still pending, however. Recently, an LFD prototype for urine POC testing based on MAB476 has been constructed, which is currently undergoing further evaluation in studies with bigger sample sizes. Overall, the evaluation of the diagnostic performance of new assays in IA remains problematic, as the vast majority of IA cases are “probable” cases and there is no established diagnostic test in clinical routine that provides sensitivity high enough to qualify the test as a reliable gold standard.

The search for reliable antigen-based POC tests for other molds, including mucorales, continues [63]. Recently, an enzyme-linked immunospot assay has been developed, for detection of Mucorales-specific T cells in peripheral blood samples [63]. Mucorales-specific T cells polarized to the production of T helper type 2 cytokines were associated with proven invasive mucormycosis and may be detected by immunoenzymatic assays or immunocytofluorimetric assays [63]. Once validated, that assay may represent a breakthrough in diagnosis of invasive mucormycosis [63].
